# A sea of connections: Reflections on connectivity from/in Oceania

**DOI:** 10.1007/s13280-022-01789-x

**Published:** 2022-10-12

**Authors:** Elodie Fache, Juliette Kon Kam King, Léa Riera, Annette Breckwoldt

**Affiliations:** 1 SENS, IRD, CIRAD, Université Paul Valery Montpellier 3, Montpellier, France; 2 Leibniz Centre for Tropical Marine Research (ZMT), Fahrenheitstrasse 6, 28359 Bremen, Germany

## Introduction

The United Nations’ First and Second World Ocean Assessments (UN 2016, 2021) alarmingly outlined the breadth of threats to the world’s oceans, spanning from biodiversity collapse to pollution (including plastics and sounds) of even its most remote and deepest areas, as well as warming and acidification. These phenomena are related to exploitative activities of unprecedented extent and intensity, combined with the contested but continuing development of coastal urbanization and (so far ‘virtual’) deep-sea mining projects, which contributes to the (present and future) alteration of oceans’ functionalities. At the same time, oceans are increasingly acknowledged for their fundamental role in supporting human societies, with much anticipation regarding their additional capacity to foster livelihoods and (blue) economies, especially given the stagnation of land-based economies and the rarefaction of terrestrial resources (OECD 2016). Essential to human wellbeing, health, and survival (Betley et al. 2021), and yet threatened, oceans are by now at the fore of both global and national political agendas as well as increasingly high on the priorities of the conservation sector, which is progressively expanding its terrestrial focus toward marine ecosystems. This is illustrated by the adoption in 2015 of the United Nations Sustainable Development Goal 14, aiming to “conserve and sustainably use the oceans, seas, and marine resources for sustainable development,”^1^ and by the commitment to protect 30% of the world’s oceans by 2030 proclaimed at the 2022 One Ocean Summit^2^ and United Nations Ocean Conference.^3^

Given that the Pacific Ocean is the largest and probably the most coveted and threatened water-body globally (Bennett et al. 2015), the region has become a central stage for imagining the future of the world’s oceans and their governance. “Ninety-eight per cent of the area occupied by Pacific Island countries and territories is ocean” (Taylor 2017: 19). For these countries and territories the ocean “is at the heart of [their] cultures,” as they “depend on it for food, income, employment, transport, and economic development” (Taylor 2017: 19), while seeing themselves as custodians “for the general welfare of all living things” (Hau’ofa 2000: 40). Consequently, many Pacific Island countries and territories have committed to the sustainable management of the large oceanic spaces and outstanding marine resources under their jurisdiction. This is best illustrated by the regional *Framework for a Pacific Oceanscape*, with its overarching vision of “a secure future for Pacific Island Countries and Territories based on sustainable development, management, and conservation of our Ocean” (Pratt and Govan 2010: 56). These countries and territories also attempt to progress their leadership in global ocean governance by drawing on their historical and fundamental connections to the Pacific Ocean, as evidenced by Fiji’s co-hosting of the United Nations Ocean Conference and launching of the Ocean Pathway in 2017, then in 2022 by the Our Ocean Conference in Palau and the Blue Climate Summit in French Polynesia, among others. This attempt faces both supporting and conflicting endeavors from international and regional organizations, other (i.e., non-Oceanian) nation-states, civil society (such as environmental NGOs), private interests (such as mining corporations), and so on, in the context of an ongoing tension between processes of ocean commoning and ocean grabbing (Fache et al. 2021).

These ocean-focused agendas integrate new concerns and perspectives for ocean governance and management. In particular, the concept of ‘integration’ has gained much ground in this field, to (re)connect different scales (e.g., global, regional, national, local policies), spaces (e.g., land and sea, inshore and offshore), temporalities (e.g., with a focus on both today’s uses and future generations’ needs), and/or stakeholders (e.g., through linkages between different sectors, such as fisheries management and biodiversity conservation, and between state and non-state actors) (Riera 2022). This movement also calls for an epistemological shift away from Western views of the world’s oceans that, just like complex systems thinking in general, “remain captive to paradigmatic assumptions that implicitly reproduce a separation between humans and nature” (West et al. 2020: 305). Rather, the integrative discourse conceptualizes relational perspectives that situate these “nodes” within continually unfolding processes and relations that “are at once what we might think of as ‘social,’ ‘ecological,’ ‘political,’ and ‘technical’” (West et al. 2020: 319).

The special section ‘Oceania: A Sea of Connections’ endeavors to contribute to this relational thinking through a focus on the Pacific Ocean and its fisheries. It emerged from the research project ‘A Sea of Connections: Contextualizing Fisheries in the South Pacific Region’ (SOCPacific, 2018–2022^4^) that sought to assess and analyze the complex web of geopolitical, policy, and socio-cultural connections within which fishing activities and fisheries management efforts occur in Oceania (Fache and Breckwoldt 2019). This collection of articles aims to highlight multi-faceted and interconnected dimensions of Pacific fisheries, beyond mere economic or ecological analyses. Among the collection’s primary objectives are the exploration and production of knowledge on the ‘sea of connections’ in which island life and livelihoods are embedded. Within this frame, the dynamics of integrative ocean governance are observed and analyzed via the lens of a wide range of participating stakeholders of South Pacific fisheries. Beyond more familiar categories of stakeholders such as fishers and administrative agents, these include researchers, local students, and children, who are themselves acting, directly or indirectly, as connectors within and between Pacific Island countries and territories. These connections include the encounter of different ways of knowing, the exchange of various forms of knowledge, and the development of new knowledge sharing pathways and networks.

Drawing on academic networks across Oceania and Europe, this special section provides a series of empirical explorations of fisheries and related spaces (e.g., reef passages), species (e.g., sea turtles), values (e.g., monetary, ecological, cultural), management instruments and processes (e.g., community-based fisheries management), and stakeholders (e.g., both state and non-state actors, inclusive of actors often neglected, like children). The juxtaposition of these case studies allows us to minimize, “on the one hand, the myopic scholarly tendency to study individual island communities in relative isolation, and on the other, homogenizing views of Oceania that arise not only from foreign clichés, but also from the unifying rhetoric of affirmative regionalist paradigms” (Looser 2015: 467). This juxtaposition also illuminates both continuities and changes in various types of overlooked (re)connections, while questioning the making of specific disconnections under the joint influence of situated practices, discourses, and knowledges. It also allows us to freshly reflect on and engage with potential reasons for processes of dis/re/connection, local adaptations to these, as well as their effects on (past, present, and future) human-ocean relations. By spotlighting such dis/re/connections, the contributing authors call for a wider attention to Oceanian socio-cosmologies, sovereignties, and norms/forms of governance to support integrated and hence connected approaches to marine management.

## Oceania as a ‘sea of islands’

A few decades ago, the Pacific scholar Epeli Hau’ofa introduced the inspiring metaphor of ‘a sea of islands’ to challenge the hegemonic belittling view of Oceania while offering a new and optimistic view of (t)his region:

“There is a world of difference between viewing the Pacific as ‘islands in a far sea’ and as ‘a sea of islands.’ The first emphasizes dry surfaces in a vast ocean far from the centers of power. Focusing in this way stresses the smallness and remoteness of the islands. The second is a more holistic perspective in which things are seen in the totality of their relationships. Continental men, namely Europeans, on entering the Pacific after crossing huge expanses of ocean, introduced the view of ‘islands in a far sea.’ […] our ancestors, who had lived in the Pacific for over two thousand years, viewed their world as ‘a sea of islands’ rather than as ‘islands in the sea.’ […] The difference between the two perspectives is reflected in the two terms used for our region: *Pacific Islands* and *Oceania*.” (Hau’ofa 1994: 152–153)

Hau’ofa highlighted that, in this renewed vision, “Oceania refers to a world of people connected to each other” (Hau’ofa 2000: 36). In other words: “What Hau’ofa has rather stressed is the connecting sea—how, despite the diversities of languages and cultures, there is an *ocean of connection* among Islanders” (Jolly 2007: 530; our emphasis).

This ocean of connection was shaped by seafarers who “were at home with the sea” (Hau’ofa 1994: 153) and progressively explored and peopled the region. Their voyaging involved social networking (e.g., through marriages), trading, and an endless circulation of knowledges, ideas, and arts (Hau’ofa 1994). Over time, “due to numerous internal and external factors, many communities throughout Oceania experienced an atrophying of long-distance voyaging ability and increased focus on land-based identities and concern” (Looser 2015: 468). Yet, since the 1970s, a voyaging revival has occurred throughout Oceania. This has involved a regional renaissance of seafaring vessels and navigational technologies, with the voyaging canoe (*vaka moana*) becoming a “primary symbol for both local sovereignty and connectivity across a considerable portion of the globe” (Looser 2015: 465).

Indeed, this ocean of connection now extends far beyond the (quite recently established national) boundaries of the South Pacific, reflecting how the Oceanian diaspora around the world has struck “roots in new resource areas, securing employment and overseas family property, expanding kinship networks through which they circulate themselves, their relatives, their material goods, and their stories” (Hau’ofa 1994: 155). As illustrated by Tongans and Samoans living in Brisbane, Australia, the Oceanian diaspora usually remains connected to its homelands, while maintaining its spiritual, familial, social, and cultural connectivity across different locations and generations; a connectivity that is expressed through ‘material cultural adaptations’ and spatial behaviors, and which is essential to individual and collective wellbeing (Faleolo 2020).

Hau’ofa’s “enlarged world of Oceania” (Jolly 2007: 532), in which the ocean stands as a “connective tissue” (Looser 2015: 468), thus connects people to each other through both ancient pasts and contemporary ties, and within as well as beyond the Pacific Ocean. It also connects people “and the sea that surrounds [their] island communities” (Hau’ofa 2000: 36):

“The ocean that surrounds us is the one physical entity that all of us in Oceania share. It is the inescapable fact of our lives. [...] All of us in Oceania today, whether indigenous or otherwise, can truly assert that the sea is our single common heritage.” (Hau’ofa 2000: 38–39)

“Many of us today are not directly or personally dependent on the sea for our livelihood; and would probably get sea-sick as soon as we set foot on a rocking boat. This means only that we are no longer sea travelers or fisherfolk, but as long as we live on our islands we remain very much under the spell of the sea; we cannot avoid it.” (Hau’ofa 2000: 37)

“[...] the sea is our pathway to each other and to everyone else, the sea is our endless saga, the sea is our most powerful metaphor, the ocean is in us.” (Hau’ofa 2000: 43)

This vision bares important implications for local-to-regional governance of the Pacific Ocean. In particular, it reframes Oceania “from the sea to the shore” (rather than the other way around; Siriwardane 2021: 73), while calling for the strengthening of a regional identity and futurity “rooted in and through a relationship to the Ocean itself, as a form of kinship” (Bambridge et al. 2021: 348).

This call is particularly significant and pressing in the face of the unequaled rush for spaces and resources that is occurring in the Pacific Ocean (Fache et al. 2021). It is a call to take the ‘Oceanian Sovereignty’ seriously, as grounded in a common heritage of the sea (Bambridge et al. 2021). This ‘Oceanian Sovereignty’ reflects an expanded notion of sovereignty, beyond the scope of “jurisdiction, rule, power, and domination as these forces are tied to a state, nation, or governing body” (West 2016: 6). This regional sovereignty may be “in tension or even opposition” with State-based sovereignty, “typically defined through rights to self-determination” (Bambridge et al. 2021: 346, 349). It touches on “control over meaning, representations, the future, ideas, and the creation of social worlds and social reproduction, as well as political control and material manifestations of control” as exercised by Indigenous and local communities (West 2016: 6). Most importantly, it focuses on these communities’ relational responsibility to the ocean and the plurality of entities therein as “partners in futurity” (Bambridge et al. 2021: 349).

“Oceanian Sovereignty […] inscribes sovereignty in the everyday enactments of *rights* of engagement, reciprocal, and relational, between individuals, communities, and island environments and ecologies as opposed to the enactments of states and their necessarily ‘elitist and westernized’ institutions of governance. This epistemological revolution […] implies that rights to exert agency over local natures rise from the ground up. Specifically, it asserts that *rights* emerge from Indigenous and local communities and will frequently exceed national borders to connect partners in a sea of islands […].” (Bambridge et al. 2021: 349; our emphasis)

Such rights are enacted, in particular, in the context of both offshore and inshore fisheries, which are central to the economy of Oceanian states and the livelihoods of their (mostly coastal) inhabitants (Gillett 2016), increasingly so since the COVID-19 pandemic (Walters et al. 2021). They are also expressed through the ever-evolving policy landscape designed to manage these fisheries (Karcher et al. 2020).

## From ‘a sea of islands’ to ‘a sea of connections’ within land-sea territories

While oceans have long been conceived in Western cultures and sciences as areas distinct from land, or even as mere voids separating the continents (Steinberg 2001), Pacific Island countries and territories consider the ocean as a continuity of land, and vice-versa. This land-sea continuum manifests itself, most importantly, in customary tenure and resource management encompassing “land-and-sea estates” (Ruddle et al. 1992: 254), while being embedded in the vernacular terms derived from the (reconstructed) Proto-Malayo-Polynesian term *banua (Chave-Dartoen 2016), such as *fenua*, *fanua*, *fonua*, *enua, whenua*, or *vanua* (Veitayaki et al. 2018). Consequently, Pacific coastal communities have often appropriated inshore areas as part of their land-sea territories (e.g., Fiji’s customary fishing rights areas or *iqoliqoli*). Since the ‘renaissance of community-based marine resource management’ at the turn of the twenty-first century (Johannes 2002), dynamic and flexible tools have been developed to manage these inshore areas,^5^ such as ‘taboo areas’ (e.g., Foale et al. 2011; Fache and Breckwoldt 2018) and Locally Managed Marine Areas (LMMAs; Govan 2009; Robertson et al. 2020). This connectivity between land and sea becomes apparent in the work by Fache et al. (2022), which highlights school-children’s views on their inseparability. The progress of ridge-to-reef approaches to environmental management seeks to better account for this land-sea inseparability, even though these approaches are often based on a ‘shore-to-sea,’ rather than a ‘sea-to-shore,’ perspective. This is described in the contribution by Fache and Pauwels (2022), which gives an overview of an informal, community-led, island-level, ridge-to-reef scheme in Fiji, involving the combination of marine closures and certified organic agriculture. The study of local perceptions and conceptualizations of the effects of this scheme points to the dynamics of relatedness and becoming between the land, the sea, and all their dwellers (human and other).

Another contribution deals with local perceptions and conceptualizations of a locally managed marine closure in Fiji’s neighboring country, Vanuatu. Pascht (2022) highlights that, in contrast to how the state or NGOs understand the term ‘conservation,’ in a coastal village of Vanuatu’s main island, ‘conservation’ is regarded by local stakeholders as a way to creatively secure, for future generations, their engagement with the sea and world-making with fish. Here again, the villagers have designed their own management practices, based on their relational ontology. Yet, in this case, these practices appear disconnected from customary marine tenure and fishing taboos, as well as from the environmental and fisheries management approaches promoted by the state and NGOs. Moreover, the local focus is not so much on the land-sea continuum, but rather on the human-(shell)fish relationships as part of a specific multispecies assemblage. This perspective is quite different from Steenbergen et al.’s (2022)**,** which draws attention to how, also in Vanuatu, community-based fisheries management innovations promoted by the Vanuatu Fisheries Department (VFD) are accepted or rejected in daily practices and across scaling phases. Their analysis of connectivity thus refers to the various social, political, and/or economic networks that local stakeholders (i.e., communities, fishers, and/or fisher groups) engage in, among other objectives, to coordinate fisheries management and ‘get things done.’ They also claim the need to move away from mosaicked landscapes of siloed projects and actor-groups, and toward an integrated national program relying on coordinated programmatic approaches.

On Moorea, French Polynesia, community-based fisheries management endeavors have given momentum to the definition, then revision, of the contested local marine spatial management plan (*Plan de Gestion de l’Espace Maritime* or PGEM). As argued by Wencélius et al. (2022), one of the contentious points was the disconnection this PGEM implied between marine and terrestrial issues, thus perpetuating the land-sea divide inherited from French colonial rule, instead of reinvigorating Polynesian understandings of the land-sea continuum and associated forms of management. These are embedded in the term *rāhui*, which refers to temporary bans on specific species or spaces. In the context of the tensions around the PGEM, the notion of *rāhui* was also used by a local association as a means of protesting against the municipal government while demanding the transfer of decision-making power to local fishers and residents, thus linking past and present, customary and political, usages of this institution. This contribution illustrates that, in Oceania, land-sea connections can be interrogated from a social-(geo)political angle by looking at how such connections are revived, redefined, or ignored through interactions and frictions between various stakeholders (e.g., administrative and state authorities, environmental and cultural activists, fishers, scientists). Such processes do not unfold only on a local level, but also at the global scale. Notably, under the United Nations Convention on the Law of the Sea, the Commission on the Limits of the Continental Shelf provides nations-states with the ability to extend their exclusive economic zones over their continental shelves, which reduces the surface of the high seas (i.e., international waters). This case suggests that land-sea connections should not only be considered from the surface, but also from the depths of the ocean and water bodies. This aspect is touched on by Breckwoldt et al. (2022a, b**)** through their focus on reef passages. Reef passages are natural or human-made breaks and channels in the coastal and fringing reefs of many Pacific islands, and their depths seem to play an important (although not yet quantified) role for their functional characteristics, as well as the activities of many of its human and non-human users (Breckwoldt et al. 2022a, b).

Breckwoldt et al. (2022a**)** call attention to the interrelated, multi-faceted, ecological, and socio-cultural connectivity that reef passages operate in within land-sea territories. Based on qualitative interviews with users (fishers, scuba divers, surfers) of these extremely important reef spaces, this study vitalizes—to our knowledge for the first time in the marine realm—the concept of ‘ecological and cultural keystone places,’ whose understanding and protection will strengthen and support both reef ecosystems and the people who depend on them. These reflections are critical today, as in many Pacific contexts, these keystone places are the subject of increasingly palpable customary claims and recognition processes (e.g., Allen et al. 2019 for New Caledonia, New Zealand and Australia). Moreover, Fache et al. (2022) show how, through their drawings and descriptions of the latter, school-children in both New Caledonia and Fiji bring to the fore the need to protect ‘ecological and cultural keystone species,’ such as sea turtles and sharks, for future generations. In parallel, Harding et al. (2022) argue for systematically taking into account ‘cultural keystone species’ in future value assessments and (co-)management strategies for coastal fisheries, in Fiji and beyond.

## Inter- and transdisciplinary approaches to ocean connectivity

The original SOCPacific project started with an interdisciplinary idea revolving around fisheries as a ‘boundary object,’ i.e., “an object which lives in multiple social worlds” (or communities of practice) and thus forms “a common boundary” between these worlds by inhabiting them simultaneously, yet with “different identities in each” (Star and Griesemer 1989: 409, 412). Fisheries indeed exhibit the main characteristics of a boundary object: “interpretive flexibility between distinctly positioned interlocutors, complexity in material and organizational structure, and sensitivity to issues of the scale or ‘granularity’ with which the object is viewed” (Mawyer 2021: 132), with “[g]roups that are cooperating without consensus” tacking back-and-forth between different forms of the object (Star 2010: 605). Moreover, fisheries are a boundary object insofar as they “build practical connections between the worlds of science and that of policy and between different knowledge domains, thus becoming devices for learning and decision making” (Mollinga 2010: S-6).

From there, the project was set out to explore to what extent these multiple worlds and knowledge domains organize—or resist, fail, etc.—to implement the increasingly widespread ‘integration’ agenda (Riera 2022). In other words, we aimed to examine what connections (practical and others) are built, as well as why and how they are established, by coalitions of stakeholders previously evolving rather separately. This requires a transdisciplinary approach, one that does not separate peoples, professionals, communities, fishers, nations, regulations, but tries to find common grounds, common objectives, overlapping responsibilities—connections and connectivity among aspects of everyday realities of marine resource users, managers, and scientists. This uncovering of a ‘sea of connections’ rather than a ‘separating sea’ is well exemplified by Kitolelei et al. (2022), whose focus on sea turtles in Fiji leads to the recommendation that both governmental and customary ways and structures could well work together for the recovery of these emblematic marine animals.

Connectivity itself has also been a particularly challenging, though valuable, boundary object for the social and natural scientists involved in this special section. Over the past decade, there has been a growing body of research investigating geo-physical, biological, and ecological connectivity within and between marine, freshwater, and terrestrial realms (e.g., in the Convention on Biological Diversity framework). The analysis of movements of marine species, sediments, nutrients, etc. from estuaries to offshore waters has increased the understanding of complex ecological processes. For instance, the movements of sea turtles, which connect feeding and breeding areas via variable and complex routes, constitute a ‘diffuse connection’ (Spotila 2004). Beyond this ecological scope, the movements of sea turtles also materialize a land-sea continuum that has a high cultural and symbolic value for Oceanians (Kitolelei et al. 2022). This value is notably highlighted by ethnoecologists and anthropologists (e.g., Sabinot and Bernard 2016), who pay attention to connections between societies and their environments, as well as between social groups, their various activities, and their shared responsibilities in local, national, or regional management frameworks and strategies. Connecting species and spaces, this value is interwoven with both inshore and offshore fisheries and with marine conservation matters and tools. In Oceania, ocean connectivity encompasses all these dimensions, with their respective schools of thoughts and notions. It does not only exist between parts that would otherwise be separate, but also between parts that together are core to a larger unit(y).

A comprehensive view of ocean connectivity (including its embodiment and enactment) therefore requires the development of inter- and transdisciplinary research approaches and methodologies. An important contribution in this respect is the study by Harding et al. (2022), where an interdisciplinary research team applies a mixed methods approach to understand the multi-faceted values of coastal fisheries on Kadavu Island, Fiji. Another contribution is the study by Fache et al. (2022), which shows that drawings are a relevant interdisciplinary tool to explore how relationships between people, the sea, and all marine life therein are understood and experienced by children, whose views are usually ignored in decision-making processes related to marine sustainability. Both contributions thus provide important insights for the design of approaches to coastal fisheries management that are more sustainable and inclusive, making communities’ ways of valuing marine species, women’s contributions to small-scale fisheries, and children’s perceptions on sustainable futures, more visible and acknowledged. Moreover, in an accelerated turn toward connections (Rodary 2019), inter- and transdisciplinary approaches and methodologies by themselves give shape to manifold connections, i.e., by bringing together knowledges from different disciplines as well as knowledges beyond academic disciplines. Such approaches are necessary to: (1) gain a more comprehensive understanding of fisheries and other phenomena around human-nature relations; (2) explore both ecological and socio-cultural values of fisheries and their implications for management and conservation; and (3) improve participation in, and ownership of, research activities by members of local and Indigenous communities.

All contributions to this special section explore ocean connectivity through the point of view of local and Indigenous communities (including children in Fache et al. 2022), national governments, regional institutions, or global conservation and ‘conservation-as-development’ (West 2006) movements. Some explore how this connectivity expands from fisheries to other sectors of activities and other practices—especially toward the social values of places and resources, farming practices, conservation policies, and national and regional ocean governance frameworks—and thereby challenge usual sectoral perspectives. Therefore, we believe that the concept of ‘friction,’^6^ as defined by Tsing (2005), is a useful lens through which to consider the manuscripts assembled in this special section. A focus on ‘friction’ allows for the study of heterogeneous, cross-cultural, partly long-distance encounters, and of “the awkward, unequal, unstable, and creative qualities of interconnection across difference” that lead to “new arrangements of culture and power,” which can be either “compromising or empowering” (Tsing 2005: 4, 6). This concept calls for specific attention to be paid to interactions and negotiations between the various stakeholders of South Pacific fisheries—for example, fishers, scuba divers, and surfers in Breckwoldt et al. (2022a), or the various individuals and organizations involved in the revision of Moorea’s local marine spatial management plan in Wencélius et al. (2022)—and to the potential culture and power reconfigurations they produce, intentionally or otherwise. This concept also sheds light on translation mechanisms of international statuses, norms and regulations at the local level (e.g., of ‘conservation’ in a village of Vanuatu in Pascht 2022), as well as of customary norms in policy frameworks (e.g., of unwritten *iTaukei* rules in better policy for sea turtle governance in Fiji in Kitolelei et al. 2022), and of what these processes produce (e.g., the emergence of a national community-based fisheries management program in Vanuatu in Steenbergen et al. 2022).

## Conclusion

Epeli Hau’ofa’s call for recognizing Oceania as ‘a sea of islands’ refers to the high degree of socialization that characterizes these marine environments; an aspect still largely underestimated in marine studies. It highlights the dense and evolving social networks that criss-cross the Pacific Ocean, including past and future generations as well as more-than-human entities (fish, spirits, places, etc.). The contributions to this special section explore this view of the connecting ocean, while also weaving together various types of ecological connectivity. This allows for a more comprehensive understanding of human-ocean relations and their potential implications for the management and conservation of spaces and species within the land-sea continuum. We therefore propose to superimpose on Hau’ofa’s view of Oceania as ‘a sea of islands’ a conceptualisation of Oceania as ‘a sea of connections,’ highlighting the multiple meanings and expressions of ocean connectivity from/in Oceania.

This ‘sea of connections’ shapes (and, in turn, is shaped by) a land-sea continuum, to be considered from both the surface and depths of the Pacific Ocean, and whose health and sustainability are essential to the well-being and future(s) of Oceanians. This land-sea continuum is home to ‘ecological and cultural keystone’ places and species that should be considered through inter- and transdisciplinary approaches to tackle their multi-faceted character. The very use of this expression, ‘a sea of connections,’ is intended to emphasize the need for an expanded notion of ‘ocean connectivity,’ one that is all at once what we might think of as geo-physical, biological, ecological, cultural, social, political, etc.

More generally, this special section outlines timely linkages between: (1) the relational turn in the social sciences and humanities as well as among some sustainability scientists (West et al. 2020); (2) the integrative turn as a new era of biodiversity conservation, supposed to merge with both sustainable development and fisheries management objectives (Riera 2022); and (3) the inter- and transdisciplinary turn, aiming to ensure that many more critical voices—academic and non-academic—are heard (Breckwoldt et al. 2021). Conceptually, we point out the relevance of articulating the notion of ‘boundary object’ with that of ‘friction’ for the analysis of the ‘sea of connections’ in Oceania. Indeed, coordination efforts for bridge-building between multiple worlds and knowledge domains necessarily involve friction that sets processes into motion and whose effects can only be partially anticipated. Finally, the various contributions also reveal, via their different themes and empirical approaches, the need to move beyond a narrow understanding of both ‘connectivity’ and ‘sovereignty,’ and to explore the relationships between these two interrelated and continually unfolding processes.

## Footnotes

1 https://www.un.org/sustainabledevelopment/oceans/.

2 https://ocean-climate.org/en/looking-back-at-the-one-ocean-summit-what-commitments-for-the-ocean/.

3 https://www.un.org/en/conferences/ocean2022.

4 https://socpacific.net/.

5 Some of which are now the focus of a movement aiming to recognize, support and report ‘Other Effective Area-based Conservation Measures’ (OECMs), see https://www.sprep.org/news/recognising-supporting-and-reporting-other-effective-area-based-conservation-measures-in-the-pacific.

6 We initially took on board this concept for investigating global changes and their drivers in the development of the SOCPacific project outline.
